# Interacting information streams on the nephron arterial network

**DOI:** 10.3389/fnetp.2023.1254964

**Published:** 2023-10-19

**Authors:** Donald J. Marsh, Anthony S. Wexler, Niels-Henrik Holstein-Rathlou

**Affiliations:** ^1^ Department of Medical Sciences, Division of Medicine and Biological Sciences, Brown University, Providence, RI, United States; ^2^ Departments of Biomedical Engineering, and Mechanical and Aerospace Engineering, University of California Davis, Davis, CA, United States; ^3^ Department of Biomedical Sciences, Panum Institute, University of Copenhagen, Copenhagen, Denmark

**Keywords:** arterial networks, nephron clusters, renal autoregulation, synchronization, partial synchronization, tubuloglomerular feedback, myogenic mechanism, frontiers

## Abstract

Blood flow and glomerular filtration in the kidney are regulated by two mechanisms acting on the afferent arteriole of each nephron. The two mechanisms operate as limit cycle oscillators, each responding to a different signal. The myogenic mechanism is sensitive to a transmural pressure difference across the wall of the arteriole, and tubuloglomerular feedback (TGF) responds to the NaCl concentration in tubular fluid flowing into the nephron’s distal tubule,. The two mechanisms interact with each other, synchronize, cause oscillations in tubular flow and pressure, and form a bimodal electrical signal that propagates into the arterial network. The electrical signal enables nephrons adjacent to each other in the arterial network to synchronize, but non-adjacent nephrons do not synchronize. The arteries supplying the nephrons have the morphologic characteristics of a rooted tree network, with 3 motifs characterizing nephron distribution. We developed a model of 10 nephrons and their afferent arterioles in an arterial network that reproduced these structural characteristics, with half of its components on the renal surface, where experimental data suitable for model validation is available, and the other half below the surface, from which no experimental data has been reported. The model simulated several interactions: TGF-myogenic in each nephron with TGF modulating amplitude and frequency of the myogenic oscillation; adjacent nephron-nephron with strong coupling; non-adjacent nephron-nephron, with weak coupling because of electrical signal transmission through electrically conductive arterial walls; and coupling involving arterial nodal pressure at the ends of each arterial segment, and between arterial nodes and the afferent arterioles originating at the nodes. The model predicted full synchronization between adjacent nephrons pairs and partial synchronization among weakly coupled nephrons, reproducing experimental findings. The model also predicted aperiodic fluctuations of tubular and arterial pressures lasting longer than TGF oscillations in nephrons, again confirming experimental observations. The model did not predict complete synchronization of all nephrons.

## Introduction

Arteries within the kidney form a demand-driven, globally connected network that distributes oxygenated blood to all nephrons. Single-nephron autoregulation of blood flow provides the demand, interactions in the network support continuous perfusion of all nephrons, and the connection to the renal artery as a single network source forms the basis for interactions among all nephrons. Each nephron regulates its blood flow by controlling the inner diameter of its afferent arteriole using a combination of two mechanisms, one sensitive to the arteriole’s transmural hydrostatic pressure difference and the other to the concentrations of Na^+^, K^+^, and Cl^−^ in tubular fluid as it flows from one renal tubular segment, the thick ascending limb of Henle’s loop, into the next, the early distal tubule. The pressure-sensitive mechanism, known as the myogenic mechanism, is a general feature of arterioles throughout the body while the concentration-sensitive mechanism, called tubuloglomerular feedback (TGF), is unique to the kidney. Epithelial transport properties of the thick ascending limbs of Henle’s loop render the ionic concentrations of the tubular fluid dependent on the fluid’s flow rate, so that TGF functions to regulate the mass flow of Na^+^, K^+^, and Cl^−^ into the distal nephron.

The macula densa, a specialized plaque of epithelial cells at the beginning of the distal tubule, is TGF’s sensor, producing a signal that reaches the vascular smooth cells of the afferent arteriole to affect the vascular contractile mechanism. Each of the mechanisms oscillates at its characteristic frequency. The mean arterial blood pressure generates a 1/f spectrum with no spectral peaks at the frequencies of the arteriolar oscillations (Marsh et al., 1990; Holstein-Rathlou et al., 1995; Wagner and Persson, 1994) suggesting that the arteriolar oscillations arise autonomously as limit cycles. Each of these two have components described mathematically with non-linear functions, and models incorporating these functions simulate the observed oscillations (Gonzalez-Fernandez and Ermentrout, 1994; Holstein-Rathlou and Marsh, 1990; Marsh et al., 2005a). The two oscillations can synchronize, forming an ensemble (Sosnovtseva et al., 2005; Sosnovtseva et al., 2007). The TGF oscillation has the longer period length of the two, and their interaction results in amplitude and frequency modulation of the myogenic mechanism by TGF Marsh et al. (2005b).

The smooth muscle controlling arteriolar diameter oscillates because of periodic changes in membrane ionic permeabilities and membrane electrical potential differences. The interaction of TGF and the myogenic mechanism generates a bimodal electrical signal that propagates along the network of arteries toward the renal artery Marsh et al. (2009). Signals from different afferent arterioles interact at arterial branch points, the interactions allowing the nephrons to synchronize their blood flows, their glomerular filtration rates, and their tubular transport functions.

The renal artery enters the kidney as a single vessel and branches at irregular intervals. The initial branches run parallel to the renal surface without giving rise to arterioles or nephrons. Branches emerge from this subsurface network and turn abruptly toward the surface, forming rooted tree networks from which afferent arterioles emerge. Each afferent arteriole enters a single glomerulus, the starting point of a nephron. The renal arterial network is thus a forest network, with individual trees running orthogonally toward the renal surface. There are several sources of variability in each tree. Terminal arteries reach their furthest extent at the surface after as few as 2 or as many as 7 branches Marsh et al. (2017). The distance separating the origins of nearest neighbor afferent arterioles varies randomly (Postnov et al., 2016; Marsh et al., 2017). The length of renal tubules is also variable. Tubular length affects the transit time from the glomerulus to the macula densa and the frequency of the TGF oscillation. Structural variability will therefore provide a variety of pathways for electrical signal propagation and interaction. Periodic withdrawal of blood from an artery into an arteriole can impose a periodicity on blood flow remaining in the artery and this rhythm can affect the dynamics of downstream nephrons. TGF, the myogenic mechanism, and rhythmic blood flow, each subject to the operation of its own underlying mechanism, constitute a triad of interacting signals that can affect tubular dynamics.

Measurements of tubular hydrostatic pressure provide reliable experimental information about nephron blood flow dynamics. The TGF and myogenic oscillations can be detected in proximal tubules (Holstein-Rathlou and Leyssac, 1986; Holstein-Rathlou, 1987; Holstein-Rathlou and Marsh, 1989; Sosnovtseva et al., 2005; Sosnovtseva et al., 2007). Oscillations from near-neighbor tubules synchronize, but oscillations from far-neighbor tubules do not (Holstein-Rathlou, 1987; Yip et al., 1992). Near-neighbor refers to nephron pairs whose afferent arterioles originate from a common artery; arterioles supplying far-neighbor nephrons arise from separate arteries. Synchronized nephron pairs constitute a nephron cluster. Because nephrons can communicate across the arterial network, we now consider the possibility that larger multi-nephron clusters can form.

Nephrons and the arterial network that supplies them provide a rich collection of interactions. We constructed a computer simulation of this system to test whether it can organize itself into clusters. This effort incorporates a nephron model that we have used singly and in pairs and adds an arterial network whose organization corresponds to patterns we found in a vascular cast of a rat kidney Marsh et al. (2017).

## Materials and methods

**FIGURE 1:**
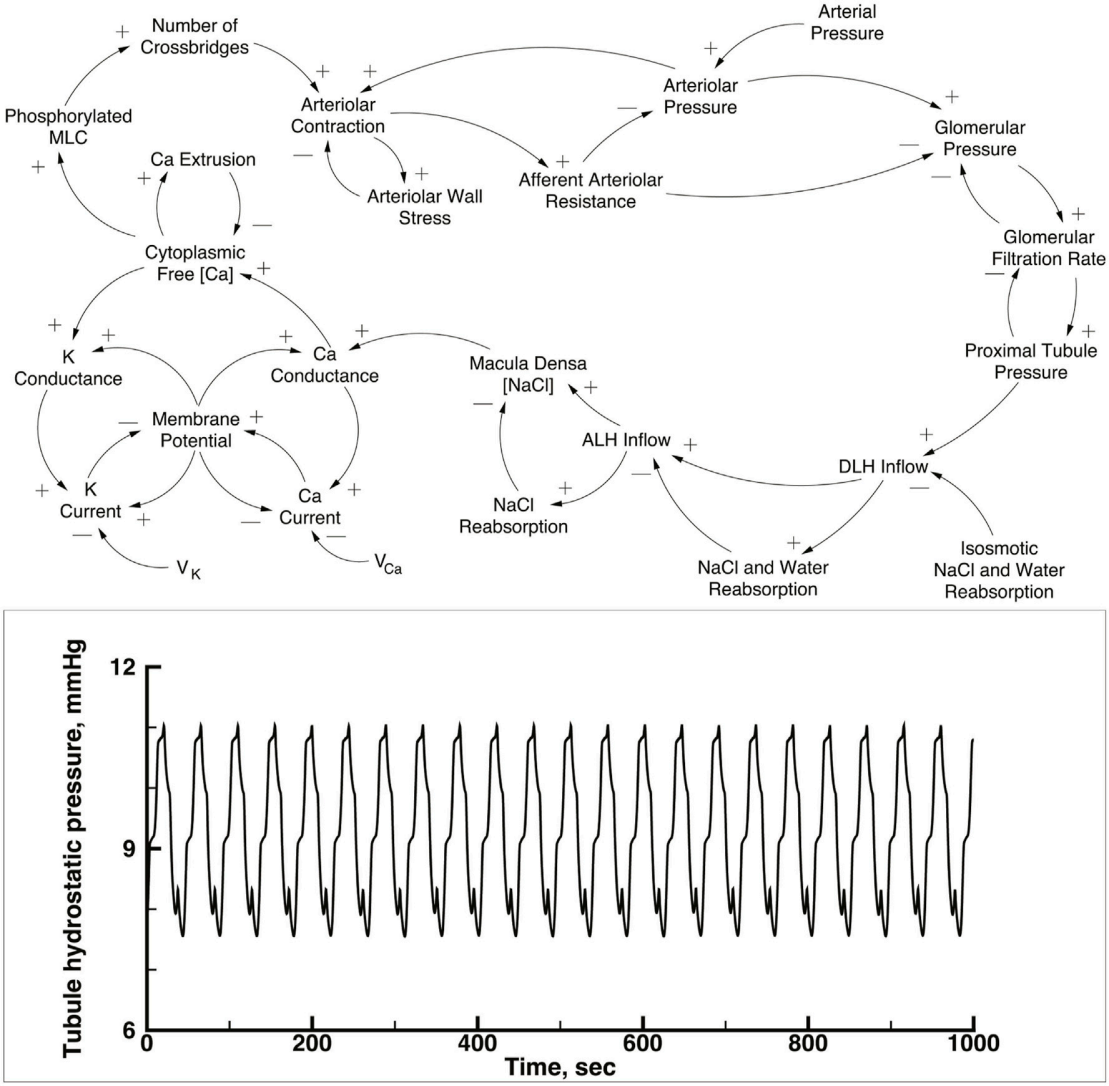
Upper panel: Causal loop diagram of a single nephron. This model was used for each of the nephron-afferent arteriole ensembles shown in Figure 2. Tubular pressure, flow rates, and NaCl concentrations were solved with a set of partial differential equations, using glomerular filtration rate, end distal tubule hydrostatic pressure, and initial NaCl concentration as boundary conditions. NaCl concentration in tubular fluid at the macula densa generates a signal that modulates *Ca* ^2+^ conductance in afferent arteriolar smooth muscle cells. Increases in the *Ca* ^2+^ current and the *K* ^+^ current produce changes in membrane potential difference of opposite sign because the reversal potential, *V*_*ca*_ and *V*_*K*_, respectively and the membrane potential have opposite signs. Membrane potential between the afferent arteriole and its adjacent vascular node (not shown in the diagram) is propagated along the vascular net of Figure 2, interacting with similar electrical signals from other nephrons. Myosin light chain abundance (MLC) varies with intracellular *Ca* ^2+^ concentration and the change in cross-bridge abundance modulates the circumference of fhe afferent arteriole. The afferent arteriole was represented with a set of initial valued ordinary differential equations. Reproduced from Laugesen et al. (2010). Lower panel: Predicted time series of tubular pressure from a 1 nephron version of the nephron-afferent arteriole model.

The simulation contains 10 copies of a single nephron model, each with a single afferent arteriole, connected to an arterial network. The nephron-arteriole model has been published (Marsh et al., 2005a; Marsh et al., 2005b; Marsh et al., 2009; Laugesen et al., 2010), and the numerical methods are the same as used in those publications. The upper panel of Figure 1 is a causal diagram of a nephron and its afferent arteriole; the equations and parameter tables are in the Supplementary Material S1. In the current work the 10 versions of the model are the same as the previously published versions except for details of how afferent arterioles connect to the arterial network, as explained below. To provide a basis for comparison with the performance of this nephron model when operating as part of the network, the lower panel shows the predicted proximal tubule pressure as a function of time in the original one nephron model.

**FIGURE 2:**
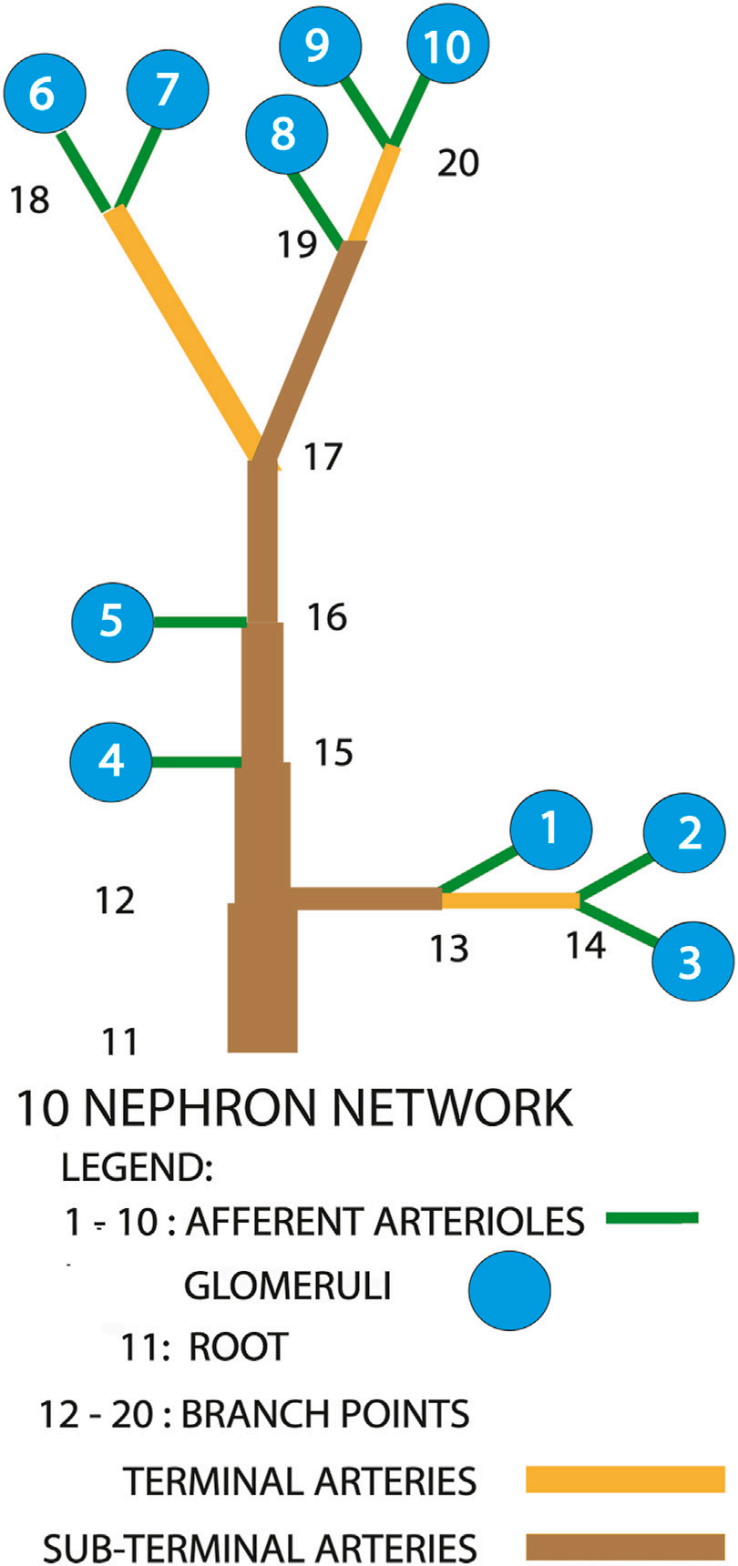
Diagram of the nephron-arterial network used for simulations. Not drawn to scale. The distribution of afferent arterioles corresponds to the distribution found in a renal vascular cast Marsh et al. (2017). Terminal arteries end in paired afferent arterioles. Sub-terminal arteries supply terminal arteries.

Figure 2 is a diagrammatic representation of the model we used. Measurements on a vascular cast of a rat kidney revealed 3 main patterns of afferent arteriolar origins Marsh et al. (2017). Fifty-three percent of all afferent arterioles arose from paired terminal arterial branches at the furthest extent of arterial trees. Nephrons 6 through 10 conform to this description and would be on or near the renal surface. Physiological measurements have been made exclusively from such nephrons. Twenty three percent of afferent arterioles arose from unpaired sub-surface arteries, and are represented in the model by nephrons 1, 2, and 3. The remaining afferent arterioles branched directly from subsurface arteries, and are shown as nephrons 4 and 5.

Each of the 10 nephron models begins with a calculation of glomerular filtration rate for that nephron by solving an ordinary differential equation for plasma protein concentration as a function of distance along an idealized glomerular capillary. The glomerular filtration rate provides an initial flow condition for the tubular model, which calculates hydrostatic pressure, flow, and NaCl concentration as functions of time and distance along the nephron. The nephrons are mechanically compliant tubules formed by epithelial cells that transport water and solutes across their walls between the lumen and the interstitial fluid. Each tubule model is divided into 3 segments - proximal tubule, descending limb of Henle’s loop, and thick ascending limb of Henle’s loop - and each is assigned epithelial transport properties identified from experimental studies. The initial boundary conditions are fluid flow rate from the calculation of glomerular filtration rate and the same NaCl concentration as in the blood plasma. The outflow fluid resistance into more distal regions of the nephron is calculated from the hydrostatic pressure at the end of the thick ascending limb of Henle’s loop and serves as a boundary condition for the model. The action of TGF is a function of the NaCl concentration at the end of the thick ascending limb and acts on the smooth muscle of the afferent arteriole to adjust its contractile mechanism.

The arterial network model is designed to represent a set of vessels that lie below the renal surface and that supply blood to the nephrons. To the best of our knowledge no observations have been reported of any of the network’s dynamic behavior from intact kidneys operating under normal physiological circumstances. We have therefore constructed the model under the assumptions that all regulatory vascular activity occurs in afferent arterioles (Carlstrom et al., 2015; Marsh et al., 2019), and that the arteries maintain constant diameters throughout the course of simulations. To provide a set of these diameters that meets the needs of the nephrons, we conducted a set of iterations of the system until the difference between successive values of the Euclidian norm of the vector of arterial nodal pressures reached an asymptote. Murray’s law Murray (1926) was used to update the radius of each branch after each iteration. Each iteration simulated 1,000 s. Once the asymptote was reached, a 4,000 s simulation was run while holding the arterial radii constant. The results from the first 1,000 s of this final simulation were used to allow additional relaxation, and the results of the final 3,000 s were recorded for analysis and presentation. All the results presented in the tables and figures are from this last 3,000 s of simulation.

The information streams that affect nephron dynamics flow through the arterial network. Blood flow is described by the Hagen Poiseuille equation, leading to the following set of equations for the pressures at the arterial nodes, under the assumption that blood flow is conserved at each node. These are the pressures at the origins of the afferent arterioles.

$$P_{N}\left(11\right)=\;\text{root pressure}\;$$ (1)

$$P_{N}\left(n\right)=P_{N}\left(m\right)-\left(\frac{8\mu L_{A}\left(m,n\right)}{\pi \left(R_{A}{\left(m,n\right)}^{4}\right)}\right)\overset{10}{\underset{i=1}{\sum }}Q_{A}\left(i\right),\;\;\;\;m=11,n=12$$ (2)

$$P_{N}\left(n\right)=P_{N}\left(m\right)-\left(\frac{8\mu L_{A}\left(m,n\right)}{\pi \left(R_{A}{\left(m,n\right)}^{4}\right)}\right)\overset{3}{\underset{i=1}{\sum }}Q_{A}\left(i\right),\;\;\;\;m=12,n=13$$ (3)

$$P_{N}\left(n\right)=P_{N}\left(m\right)-\left(\frac{8\mu L_{A}\left(m,n\right)}{\pi \left(R_{A}{\left(m,n\right)}^{4}\right)}\right)\overset{3}{\underset{i=2}{\sum }}Q_{A}\left(i\right),\;\;\;\;m=13,n=14$$ (4)

$$P_{N}\left(n\right)=P_{N}\left(m\right)-\left(\frac{8\mu L_{A}\left(m,n\right)}{\pi \left(R_{A}{\left(m,n\right)}^{4}\right)}\right)\left(\overset{10}{\underset{i=1}{\sum }}Q_{A}\left(i\right)-\overset{3}{\underset{j=1}{\sum }}Q_{A}\left(j\right)\right),\;m=12,n=15$$ (5)

$$P_{N}\left(n\right)=P_{N}\left(m\right)-\left(\frac{8\mu L_{A}\left(m,n\right)}{\pi \left(R_{A}{\left(m,n\right)}^{4}\right)}\right)\left(\overset{10}{\underset{i=1}{\sum }}Q_{A}\left(i\right)-\overset{4}{\underset{j=1}{\sum }}Q_{A}\left(j\right)\right),\;m=15,n=16$$ (6)

$$P_{N}\left(n\right)=P_{N}\left(m\right)-\left(\frac{8\mu L_{A}\left(m,n\right)}{\pi \left(R_{A}{\left(m,n\right)}^{4}\right)}\right)\left(\overset{10}{\underset{i=1}{\sum }}Q_{A}\left(i\right)-\overset{5}{\underset{j=1}{\sum }}Q_{A}\left(j\right)\right),\;m=16,n=17$$ (7)

$$P_{N}\left(n\right)=P_{N}\left(m\right)-\left(\frac{8\mu L_{A}\left(m,n\right)}{\pi \left(R_{A}{\left(m,n\right)}^{4}\right)}\right)\overset{7}{\underset{i=6}{\sum }}Q_{A}\left(i\right),\;\;\;\;m=17,n=18$$ (8)

$$P_{N}\left(n\right)=P_{N}\left(m\right)-\left(\frac{8\mu L_{A}\left(m,n\right)}{\pi \left(R_{A}{\left(m,n\right)}^{4}\right)}\right)\overset{10}{\underset{i=8}{\sum }}Q_{A}\left(i\right),\;\;\;\;m=17,n=19$$ (9)

$$P_{N}\left(n\right)=P_{N}\left(m\right)-\left(\frac{8\mu L_{A}\left(m,n\right)}{\pi \left(R_{A}{\left(m,n\right)}^{4}\right)}\right)\overset{10}{\underset{i=9}{\sum }}Q_{A}\left(i\right),\;\;\;\;m=19,n=20$$ (10)

*P*_*N*_(*m*) and $P(\left.n\right)$ are the arterial pressures in the *m*th and *n*th nodes, repectively, of Figure 2. The root pressure was 85 mmHg in all simulations, a value chosen to approximate the local arterial pressure when the mean pressure in the aorta is 100 mmHg. *L*_*A*_ (*m*, *n*) and *R*_*A*_ (*m*, *n*) are the lengths and radii, respectively, of the arterial segments beginning at arterial nodes *m* and ending at *n*, *Q*_*A*_(*i*) is the blood flow into the arteriole of the *i*th nephron, and *μ* is blood viscosity.

The arterial network also serves as the pathway for electrical signals originating in the afferent arterioles. The TGF signal originates in the macula densa and passes through several cells and intercellular spaces before reaching the afferent arteriole, having been transformed into a chemical signal in the process. The final signal likely binds to the afferent arteriole at or near its entrance into the glomerulus. The effect of this binding site location is that the signal is not applied uniformly over the length of the afferent arteriole. To account for this localizing effect we divided the afferent arteriole into 2 coupled segments of unequal length, each receiving a different fraction of the TGF signal. All other functions of the 2 arteriolar segments were identical. In the original version of the nephron model the second segment of the afferent arteriole was connected to ground through an electrical resistance specified by a single parameter. In the current work that connection is to the arterial network at a vascular node, and the current pathway to ground is through the network.

To calculate the distribution of electrical signals we modeled the arterial network as a linear bridge resistor with 10 nephrons; Figure 2 is its diagrammatic representation. Twelve non-linear ordinary differential equations comprise the set of model equations describing the dynamics of each afferent arteriole. This model is based on the work of Gonzalez-Fernandez and Ermentrout who simulated the dynamics of small cerebral arteries Gonzalez-Fernandez and Ermentrout (1994). The model used in our work was modified to include an action of TGF. The 120 equations for all afferent arterioles were solved simultaneously at each time step, and provided the values of the transmembrane electrical potential differences. Electrical potentials imposed by afferent arterioles at the periphery of the arterial network uniquely determine the potentials on each of the internal nodes. These internal node voltages are calculated from *ν*_**unknown**_ = **−D** ^**−1**^ **C** *ν*_**known**_, where **C** is an 11 × 9 matrix, and **D** is a symmetric 9 × 9 matrix; *ı* designates columns and *ȷ* rows in both matrices. The **C**_*ı*,*ȷ*_ are the conductances between nodes on the periphery, *ı*, and those in the interior, *ȷ*, the off-diagonal terms, **D**_*ı*,*ȷ*_ are the conductances among the interior nodes. The diagonal terms, **D**_*ı*,*ı*_ are the negative of the sum over all of the conductances in row *ı* of **C** and **D**; *ν*_**known**_ is the vector of known electrical potentials; and *ν*_**unknown**_ is the vector of unknown electrical potentials. The 11 elements of *ν*_**known**_ are the electrical potentials in each of the 10 afferent arterioles and the electrical ground. The elements of *ν*_**unknown**_ are the electrical potentials of the 9 vascular nodes. The quantity **D** ^**−1**^ **C** is only dependent on network topology which does not change throughout each simulation. The conductance of each arterial segment is the product of the conductivity and the length, both of which remained constant during the course of a simulation, and the radius, and was adjusted after each iteration. Conductances of each arterial segment were assumed proportional to the circumference of the segment, and inversely proportional to its length. $g_{m,n}=g_{A}2\pi R_{A}\left(m,n\right)/L(\left.m,n\right)$ , where $g(\left.m,n\right)$ is the conductance of the arterial segment connecting the mth and nth nodes, and *g*_*A*_ is the conductivity of the arterial wall.

Renal tubules vary in length, and the distances separating the origins of their afferent arterioles follow a Poisson distribution (Postnov et al., 2016; Marsh et al., 2017). Although no distance measures accompanied reports of tubule pressure measurements, each experiment is likely to have been conducted in a unique structure, and the simulations were designed to capture this variability. The process that produces the time varying TGF signal at the macula densa depends on a fluid wave originating with glomerular filtration. The wavelength in turn depends on the nephron length. We used a random number generator to assign lengths of nephrons and arterial segments. Each simulation received a unique seed. Tubule lengths were 1.95, 1.97 1.99, and 2.1 cm. The patterns of arterial network topology and the lengths of individual segments were based on measurements of a vascular cast of a rat kidney (Marsh et al., 2017). The length of each arterial segment was varied with a random number generator to assign the length a value in the interval [75%,125%] of the measured mean value.

### Data analysis

We sought to determine whether signals generated by the periodic activity of renal afferent arterioles produce interactions that lead to nephron cluster formation. Clusters are defined by synchronization of rhythmic activity. Each afferent arteriole generates a bimodal electrical signal from the interacting operations of TGF and the myogenic mechanism, and interacts with similar but not necessarily identical bimodal signals from other afferent arterioles. We began the analytical process by applying complex demodulation to each simulated time series, a process that separated the bimodal signal into high (myogenic)- and low (TGF)-frequency components.

The dispersion of the phase angle difference over time is a measure of the extent of synchronization of 2 processes, and its mean value characterizes the type of synchronization Mormann et al. (2000). Application of their method requires techniques of directional statistics to provide these estimates from simulation results. Simulations provided time series of proximal tubule hydrostatic pressure sampled at 4/s. After complex demodulation, mean values were subtracted from each individual value of a given pair of time series, yielding 2 real-valued processes, *x*(*k*) and *y*(*k*), 1 ≤ *k* ≤ *N*, *N* = the number of sample points Berens (2009). The instantaneous phase of a real-valued discrete process, *x*(*k*) can be defined as the argument of the corresponding analytic signal, $\overset{∼}{x}$ , which is complex valued and given by $\overset{∼}{x}=x\left(k\right)+i\cdot H\left[x\left(k\right)\right]$ , where H[x(k)] is the Hilbert transform of *x*(*k*) and *i* is the imaginary unit Vakman (1998). The instantaneous phase difference between two processes, Δ*θ*(*k*), is the argument of the complex number $\overset{∼}{z}\left(k\right)$ , where $\overset{∼}{z}\left(k\right)=\frac{\overset{∼}{y}\left(k\right)}{\overset{∼}{x}\left(k\right)}$ , 1 ≤ *k* ≤ *N*. The mean resultant vector, $\bar{\rho }$ , is given by $\bar{\rho }=\frac{1}{N}\sum_{n=1}^{N}\frac{\overset{∼}{z}\left(k\right)}{\left|\overset{∼}{z}\left(k\right)\right|}$ . $\bar{\rho }$ is a complex number that lies on or inside the unit circle in the complex plane; $\left|\bar{\rho }\right|$ is its length. Because we are calculating the phase angle difference, the length of the mean resultant vector will be 1 if the two processes are phase locked, but 0 if they are completely desynchronized because then the phase angle differences are distributed uniformly on the unit circle. We designate $\left|\bar{\rho }\right|$ as the synchronization metric. A high value of $\left|\bar{\rho }\right|$ reflects strong interaction between the nephrons in the pair, and that they are synchronized or close to synchrony. The argument of $\bar{\rho }$ is the mean phase angle difference, $\bar{\Delta \phi }$ . This analytic procedure generated estimates of myogenic-myogenic and TGF-TGF synchronization in nephron pairs, and was applied to all 45 possible nephron interactions in the 10 nephron set.

## Results

To validate the single nephron - afferent arteriole model we used experimental results collected from surface nephrons of intact kidneys of anesthetized young adult male rats (Holstein-Rathlou and Leyssac, 1986; Holstein-Rathlou, 1987; Holstein-Rathlou and Marsh, 1989; Yip et al., 1992; Yip et al., 1993). These experimental results are time series of hydrostatic pressure measured in proximal tubules on the surface of the kidneys. The nephron - afferent arteriolar model has been implemented in one- and two-nephron versions (Marsh et al., 2005a; Marsh et al., 2005b; Marsh et al., 2009; Laugesen et al., 2010). The coefficient representing the TGF-myogenic coupling is a bifurcation parameter for this model, with negative Lyapunov exponents for the parameter set we used in the present studyLaugesen et al. (2010). These earlier versions of the model also predicted an interaction between TGF and the myogenic mechanism that produced frequency and amplitude modulation of the myogenic mechanism by TGF. Analysis of experimental data with wavelet transforms confirmed these predictions (Marsh et al., 2005a; Marsh et al., 2009; Sosnovtseva et al., 2005).

Figure 1, lower panel, displays predicted hydrostatic pressure in a single nephron implementation of the model. This figure is intended to provide a comparison with the behavior of the same model in the multi-nephron configuration of Figure 2. Each of the simulations presented below were run with a constant arterial pressure of 85 mmHg at node 11. This choice was based on the assumption of a mean arterial blood pressure in the aorta of 100 mmHg, with pressure drops from the aorta to the renal artery through a series of branches proceeding toward the renal surface.

**FIGURE 3:**
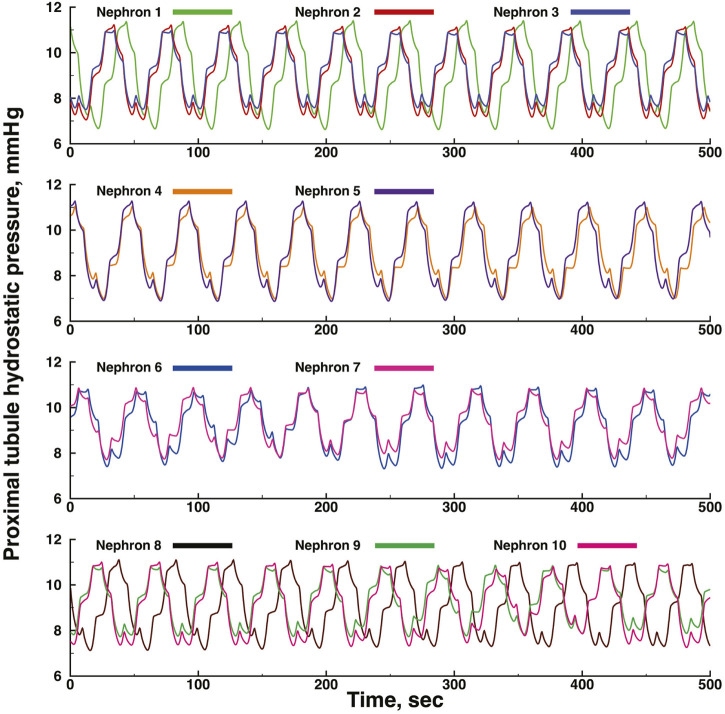
Time series of proximal tubule pressures in a run of the model shown in Figure 2.

**FIGURE 4:**
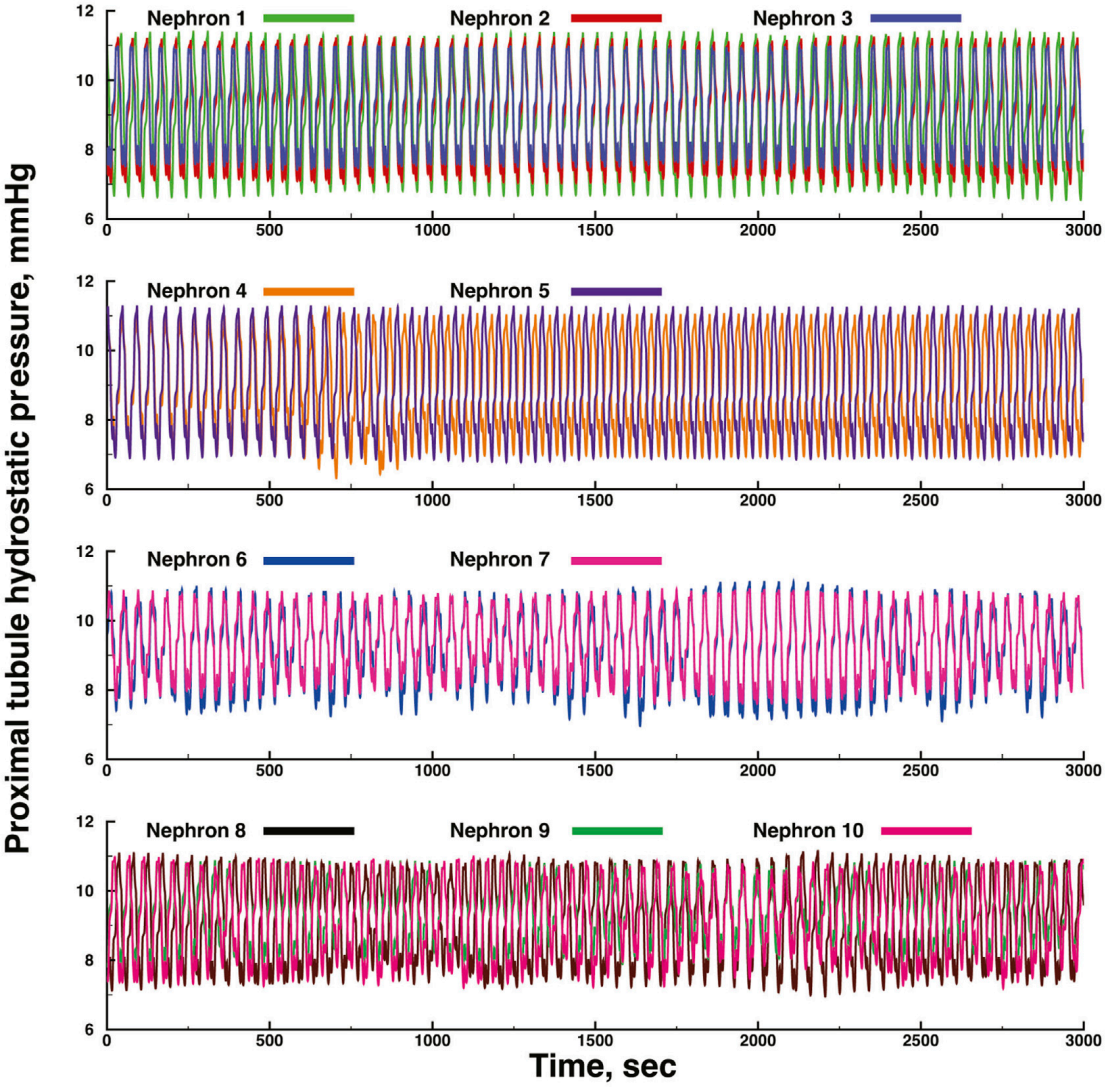
Time series of proximal tubule pressures in a run of the same model shown in Figure 3, extended to a duration of 3,000s.

**FIGURE 5:**
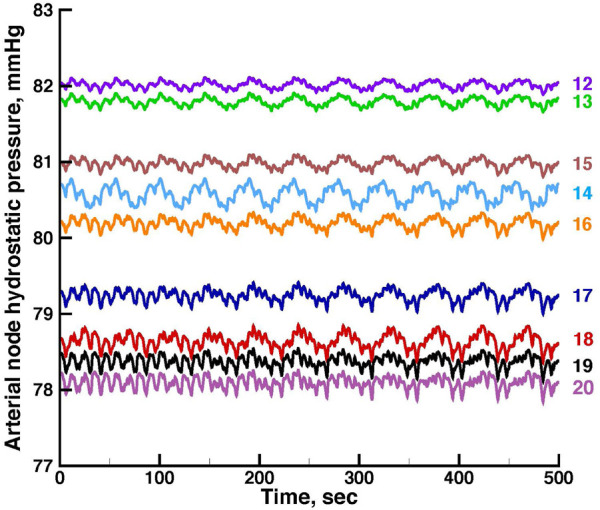
Time series of vascular hydrostatic pressures in the same run shown in Figure 3. Numbers at the right refer to the nodes in Figure 2. Pressure in node 11 was 85 mmHg throughout the simulation.

**FIGURE 6:**
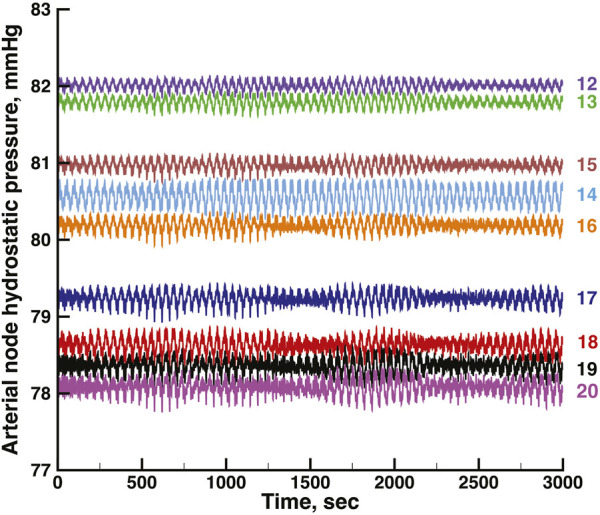
Time series of vascular hydrostatic pressures in the same run shown in Figure 4. Numbers at the right refer to the nodes in Figure 2. Pressure in node 11 was 85 mmHg throughout the simulation.

Figures 3, 4 show time series of predicted tubule pressures in each of 10 nephrons, using the configuration depicted in Figure 2. Figure 4 displays the entire run. Figures 5, 6 show the pressures in each of the 9 arterial nodes from the same simulation. Figures 3, 5 show the first 500 s of the simulation, a duration chosen to provide resolution sufficient to visualize the details of individual time series. The model contains 3 nephron pairs whose afferent arterioles originate at the end of terminal arteries: nephrons 2 and 3, 6 and 7, and 9 and 10. Each pair originates from a single point in the arterial segment supplying them, examples of strong coupling. The unpaired nephrons are separated from their neighboring nephrons by lengths of arterial segments; interposing a non-active arterial segment creates weak coupling. Nephron 1 is adjacent to nephrons 2 and 3 but separated from them by an arterial segment; nephron 8 has a similar relationship to nephrons 9 and 10. Nephrons 4 and 5 arise separately from a sub-terminal artery, but are not adjacent to any of the 3 nephron pairs. The identical configuration was used in 15 simulations, with the set of nephron and arterial lengths determined by a random number generator, each of the 15 simulations receiving a unique seed value and producing a unique set of results. The results in Figures 3, 4 are from a single simulation selected because it exhibits an extensive repertoire of the kinds of behaviors we found to a greater or lesser extent in the other 14 simulations. All simulations were run for a total of 3,000 s. Tables 1-3 contain the synchronization measures for TGF, the myogenic mechanism, and the arterial nodal pressures, respectively. Table 4 provides the same measures for the interactions between pressures in the proximal tubules and in the arterial segment supplying blood to the nephron.

**TABLE 1:** Synchronization measures for the TGF interactions among 10 nephrons. In each of 15 simulations a random number generator with a unique seed assigned values of the lengths of nephrons and arterial segments. The cells in the tables shown with a light blue background are from nephrons whose afferent arterioles are paired directly with no intervening arterial segments.

	**A**.: Synchronization metric, $\left|\bar{\rho }\right|$ , mean ± SD, for TGF dynamics								
Nephron	2	3	4	5	6	7	8	9	10
1	0.78 ± 0.23	0.78 ± 0.23	0.72 ± 0.24	0.79 ± 0.19	0.41 ± 0.27	0.41 ± 0.27	0.38 ± 0.22	0.43 ± 0.24	0.42 ± 0.24
2		0.99 ± 0.01	0.72 ± 0.29	0.76 ± 0.27	0.51 ± 0.26	0.51 ± 0.26	0.50 ± 0.24	0.45 ± 0.26	0.45 ± 0.26
3			0.72 ± 0.28	0.76 ± 0.27	0.51 ± 0.26	0.51 ± 0.26	0.50 ± 0.24	0.45 ± 0.26	0.45 ± 0.26
4				0.74 ± 0.34	0.46 ± 0.25	0.46 ± 0.25	0.49 ± 0.26	0.52 ± 0.30	0.52 ± 0.30
5					0.52 ± 0.23	0.53 ± 0.23	0.50 ± 0.29	0.45 ± 0.27	0.45 ± 0.27
6						0.99 ± 0.01	0.60 ± 0.34	0.59 ± 0.25	0.59 ± 0.25
7							0.59 ± 0.34	0.58 ± 0.25	0.59 ± 0.25
8								0.62 ± 0.26	0.62 ± 0.26
9									0.98 ± 0.02

	**B**. Phase angle difference, $\bar{\Delta \phi }$ , mean ± SD, for TGF dynamics								
Nephron	2	3	4	5	6	7	8	9	10
1	2.13 ± 1.06	2.13 ± 1.05	1.55 ± 1.06	1.77 ± 0.97	1.60 ± 0.98	1.58 ± 0.97	1.15 ± 1.02	2.07 ± 0.81	2.06 ± 0.79
2		0.12 ± 0.09	1.81 ± 0.73	1.89 ± 0.94	2.05 ± 0.93	2.05 ± 0.99	2.01 ± 0.97	1.31 ± 1.13	1.25 ± 1.00
3			1.78 ± 0.74	1.94 ± 0.94	2.02 ± 0.95	2.02 ± 0.99	1.95 ± 0.86	1.38 ± 1.11	1.30 ± 1.00
4				1.92 ± 0.68	1.75 ± 0.92	1.73 ± 0.82	1.56 ± 0.82	1.94 ± 0.93	1.91 ± 1.00
5					1.75 ± 0.72	1.71 ± 0.78	1.35 ± 0.95	1.58 ± 1.04	1.65 ± 1.08
6						0.13 ± 0.15	1.77 ± 0.67	2.17 ± 0.46	2.28 ± 0.44
7							1.88 ± 0.70	2.07 ± 0.56	2.19 ± 0.54
8								2.44 ± 0.76	2.42 ± 0.81
9									0.15 ± 0.13

**TABLE 2:** Synchronization measures for the myogenic interactions among 10 nephrons. In each of 14 simulations a random number generator with a unique seed assigned values of tubule length. The cells in the tables shown with a light blue background are from nephrons whose afferent arterioles are paired directly with no intervening arterial segments.

	**A**.: Synchronization metric, $\left|\bar{\rho }\right|$ , mean ± SD, for myogenic dynamics								
Nephron	2	3	4	5	6	7	8	9	10
1	0.13 ± 0.08	0.13 ± 0.08	0.12 ± 0.12	0.10 ± 0.06	0.05 ± 0.03	0.05 ± 0.04	0.06 ± 0.04	0.05 ± 0.07	0.05 ± 0.06
2		0.95 ± 0.04	0.16 ± 0.11	0.19 ± 0.10	0.06 ± 0.04	0.06 ± 0.04	0.08 ± 0.08	0.08 ± 0.08	0.08 ± 0.07
3			0.16 ± 0.12	0.19 ± 0.10	0.06 ± 0.05	0.06 ± 0.05	0.08 ± 0.07	0.08 ± 0.07	0.08 ± 0.07
4				0.26 ± 0.17	0.06 ± 0.04	0.06 ± 0.04	0.05 ± 0.03	0.08 ± 0.06	0.08 ± 0.06
5					0.08 ± 0.06	0.07 ± 0.06	0.06 ± 0.04	0.04 ± 0.03	0.04 ± 0.03
6						0.97 ± 0.03	0.20 ± 0.14	0.12 ± 0.08	0.12 ± 0.08
7							0.20 ± 0.14	0.13 ± 0.08	0.13 ± 0.08
8								0.13 ± 0.10	0.13 ± 0.10
9									0.97 ± 0.03

	**B**. Phase angle difference, $\bar{\Delta \phi }$ , mean ± SD, for myogenic dynamics								
Nephron	2	3	4	5	6	7	8	9	10
1	1.35 ± 0.78	1.18 ± 0.70	1.69 ± 0.88	1.59 ± 0.97	1.72 ± 0.90	1.63 ± 0.84	1.67 ± 0.71	1.83 ± 0.84	1.72 ± 0.95
2		0.02 ± 0.03	1.77 ± 1.11	1.67 ± 1.07	1.61 ± 1.06	1.59 ± 1.03	1.51 ± 0.78	1.95 ± 0.65	1.99 ± 0.67
3			1.62 ± 1.09	1.59 ± 1.03	1.67 ± 1.14	1.65 ± 1.14	1.43 ± 0.90	2.02 ± 0.68	2.03 ± 0.70
4				1.58 ± 1.02	1.82 ± 1.09	1.81 ± 1.02	1.51 ± 0.83	1.49 ± 1.10	1.58 ± 1.03
5					1.56 ± 0.97	1.56 ± 1.00	1.61 ± 1.09	2.09 ± 0.65	1.73 ± 0.84
6						0.01 ± 0.01	1.57 ± 0.92	1.82 ± 0.86	1.73 ± 0.87
7							1.53 ± 0.87	1.87 ± 0.84	1.77 ± 0.90
8								1.28 ± 0.97	1.30 ± 0.99
9									0.01 ± 0.01

**TABLE 3:** Synchronization measures for interactions in hydrostatic pressures among the arterial nodes of the network.

**A**.: Synchronization metric, $\left|\bar{\rho }\right|$ , mean ± SD, for TGF interactions								
Node	13	14	15	16	17	18	19	20
12	0.92 ± 0.17	0.73 ± 0.25	0.93 ± 0.11	0.84 ± 0.18	0.78 ± 0.22	0.70 ± 0.26	0.73 ± 0.19	0.73 ± 0.17
13		0.76 ± 0.23	0.82 ± 0.18	0.73 ± 0.22	0.68 ± 0.24	0.62 ± 0.28	0.69 ± 0.21	0.65 ± 0.20
14			0.65 ± 0.24	0.59 ± 0.24	0.54 ± 0.24	0.55 ± 0.19	0.53 ± 0.22	0.53 ± 0.21
15				0.93 ± 0.13	0.86 ± 0.19	0.77 ± 0.22	0.82 ± 0.17	0.80 ± 0.16
16					0.94 ± 0.15	0.78 ± 0.21	0.90 ± 0.14	0.87 ± 0.13
17						0.87 ± 0.14	0.90 ± 0.18	0.87 ± 0.16
18							0.75 ± 0.22	0.71 ± 0.22
19								0.95 ± 0.11

**B**. Phase angle differences, $\bar{\Delta \phi }$ , mean ± SD, for TGF interactions								
Node	13	14	15	16	17	18	19	20
12	0.07 ± 0.07	0.45 ± 0.45	0.13 ± 0.11	0.24 ± 0.20	0.30 ± 0.24	0.44 ± 0.35	0.31 ± 0.24	0.33 ± 0.26
13		0.36 ± 0.32	0.21 ± 0.18	0.34 ± 0.27	0.39 ± 0.31	0.60 ± 0.47	0.40 ± 0.31	0.42 ± 0.33
14			0.72 ± 0.64	0.96 ± 0.71	1.04 ± 0.75	1.27 ± 0.92	1.05 ± 0.64	1.02 ± 0.63
15				0.12 ± 0.07	0.17 ± 0.10	0.31 ± 0.20	0.18 ± 0.14	0.21 ± 0.16
16					0.07 ± 0.06	0.20 ± 0.15	0.12 ± 0.09	0.17 ± 0.09
17						0.15 ± 0.14	0.08 ± 0.06	0.15 ± 0.08
18							0.28 ± 0.20	0.37 ± 0.19
19								0.07 ± 0.04

**C**.: Synchronization metric, $\left|\bar{\rho }\right|$ , mean ± SD, for myogenic interactions								
Node	13	14	15	16	17	18	19	20
12	0.89 ± 0.22	0.76 ± 0.14	0.93 ± 0.15	0.85 ± 0.19	0.77 ± 0.21	0.77 ± 0.17	0.73 ± 0.20	0.75 ± 0.17
13		0.74 ± 0.22	0.80 ± 0.23	0.72 ± 0.24	0.66 ± 0.23	0.65 ± 0.21	0.69 ± 0.19	0.63 ± 0.20
14			0.70 ± 0.06	0.61 ± 0.09	0.55 ± 0.12	0.54 ± 0.09	0.51 ± 0.11	0.51 ± 0.08
15				0.93 ± 0.16	0.87 ± 0.20	0.85 ± 0.13	0.82 ± 0.20	0.84 ± 0.14
16					0.94 ± 0.15	0.85 ± 0.20	0.90 ± 0.16	0.92 ± 0.03
17						0.91 ± 0.16	0.89 ± 0.22	0.91 ± 0.14
18							0.79 ± 0.23	0.81 ± 0.19
19								0.94 ± 0.18

**D**. Phase angle differences, $\bar{\Delta \phi }$ , mean ± SD, for myogenic interactions								
Node	13	14	15	16	17	18	19	20
12	0.10 ± 0.23	0.08 ± 0.18	0.05 ± 0.15	0.11 ± 0.22	0.18 ± 0.32	0.14 ± 0.31	0.16 ± 0.24	0.12 ± 0.22
13		0.16 ± 0.28	0.15 ± 0.26	0.20 ± 0.29	0.28 ± 0.35	0.24 ± 0.35	0.18 ± 0.27	0.22 ± 0.30
14			0.06 ± 0.04	0.11 ± 0.14	0.17 ± 0.26	0.14 ± 0.23	0.15 ± 0.18	0.13 ± 0.15
15				0.06 ± 0.19	0.13 ± 0.31	0.09 ± 0.26	0.12 ± 0.23	0.08 ± 0.19
16					0.08 ± 0.27	0.15 ± 0.34	0.06 ± 0.16	0.02 ± 0.02
17						0.08 ± 0.23	0.13 ± 0.30	0.09 ± 0.27
18							0.21 ± 0.35	0.17 ± 0.33
19								0.05 ± 0.18

**TABLE 4:** Synchronization measures for interactions in hydrostatic pressures between individual tubules and the arterial nodes from which their afferent arterioles arise. A: Synchronization metric, $\left|\bar{\rho }\right|$ , mean ± SD, for TGF interactions; B: Phase angle differences, $\bar{\Delta \phi }$ , mean ± SD, for TGF interactions; C: Synchronization metric, $\left|\bar{\rho }\right|$ , mean ± SD, for myogenic interactions; and D: Phase angle differences, $\bar{\Delta \phi }$ , mean ± SD, for myogenic interactions.

Nephron	Node	A	B	C	D
1	13	0.56±0.27	1.47±0.94	0.21±0.08	1.85±0.79
2	14	0.97±0.03	2.81±0.20	0.70±0.06	2.12±0.06
3	14	0.97±0.02	2.76±0.23	0.70±0.05	2.13±0.09
4	15	0.60±0.25	1.64±0.88	0.22±0.09	2.09±0.41
5	16	0.60±0.23	2.12±0.80	0.23±0.10	2.15±0.42
6	18	0.74±0.23	2.47±0.41	0.56±0.10	1.92±0.41
7	18	0.74±0.23	2.56±0.37	0.56±0.10	1.92±0.42
8	19	0.57±0.23	1.49±0.93	0.30±0.10	2.0±10.66
9	20	0.75±0.19	2.47±0.57	0.57±0.11	1.94±0.32
10	20	0.75±0.20	2.47±0.63	0.56±0.11	1.93±0.32

The time series for nephrons 2 and 3 oscillate at identical frequencies for both TGF and the myogenic oscillations. The amplitudes differ because the 2 nephrons were assigned different lengths. Five smaller oscillations appear during each of the longer, larger TGF oscillations, illustrating the 5:1 synchronization ratio found experimentally (Sosnovtseva et al., 2005; Sosnovtseva et al., 2007). The oscillations produced by the simulation of Nephron 1 do not have exactly the same frequencies as those of nephrons 2 and 3, indicating that nephron 1 has not fully synchronized with the other 2. Nephrons 4 and 5 exhibit time series similar to each other for the first 600 s, but then a phase difference appears between them and grows over the next 400 s. Since the models for these nephrons would generate stable limit cycle oscillations, we conclude that the change emerging at 600 s is a result of interactions with other elements in the network. The time series produced by nephrons 6 and 7 have variable amplitudes, but the myogenic:TGF ratios do not change and both oscillations remain fully synchronized. Despite the fact that the 2 afferent arterioles originate as a pair with no arterial segment separating them, the variability of the oscillation amplitudes likely reflects interactions with other elements in the network. Nephrons 8, 9, 10 have a spatial relationship similar to that of nephrons 1,2, and 3. The oscillations of nephrons 9 and 10 are fully synchronized but nephron 8 is not in phase with those of 9 and 10, and the phase angle difference between 8 and the other two varies during the course of the simulation.

None of the time series in Figure 2 through 5 is periodic. The effect is more prominent in some of the nephrons than in others. Each of the 15 simulations showed these low frequency fluctuations, but the distribution among nephrons varied from one simulation to the next. The single nephron result shown in Figure 1 remains stable indefinitely, indicating once again that the low frequency fluctuations arise from interactions in the network. The lower frequency fluctuations are seen more clearly in Figures 4, 5.

Figures 5, 6 show time series of the blood pressure in each of the arterial nodes of the network, for the same time intervals in Figures 3, 4, respectively. The pressure at the network root, node 11, is a constant 85 mmHg. The pressure fluctuations arise because of the periodic withdrawal of blood into each afferent arteriole. The pressure drop over the extent of the network was approximately 4 mmHg, and the fluctuation amplitudes were less than 1 mmHg. Blood flowing through a network will necessarily require a pressure difference, the consequence of which is that except for the 2 nephrons of each pair, origins of the other afferent arterioles do not experience identical arterial blood pressures and must be able to exercise local regulation of their arteriole’s hydraulic conductance. The variety of arterial pressures among the afferent arterioles has the potential for affecting the network electrical signalling.

Tables 1, 2 contain the averages and standard deviations of the 2 measures, $\left|\bar{\rho }\right|$ and $\bar{\Delta \phi }$ . For these and subsequent tables, 15 simulations were run using a random number generator to determine the lengths of nephrons and of arteries, each run using a unique seed value. Ten nephrons generate 45 interactions at each of 2 frequencies. The synchronization metrics were calculated for each interaction, and the average values and standard deviations for each of the 90 interactions in each of the 15 simulations are presented in the tables. Table 1 shows complete synchronization of the TGF oscillations of each of the nephron pairs 2 v 3, 6 v 7, and 9 v 10. The values of $\left|\bar{\rho }\right|$ for all other tubular interactions fell short of reaching the unit circle, and the phase angle differences were dispersed. A similar pattern of results were found for the myogenic oscillation.

The average value of the synchronization metric is 0.99 for the TGF oscillation in the nephron 2 v. 3, 6 v. 7, and 9 v. 10 interactions, indicating full synchronization in each pair. The phase angle differences for this group of nephron pairs averaged 0.13 radians, small deviations from 1.0 to 0.0, arising from the differences in nephron lengths. These 3 nephron pairs share the property that their electrical interactions are directly from one afferent arteriole to another, and do not involve an intermediate length of artery. Interactions of nephrons 1, 4, 5, and 8 with other nephrons include arterial segments as part of their signal pathways. The synchronization metrics for these 4 unpaired nephrons with any other nephrons have smaller values and larger phase angle differences than are generated by the strongly coupled nephrons. The 3 nephron pairs are separated from each other by arterial segments, and the synchronization metrics and phase angle differences between different nephron pairs are similar to those generated by the unpaired nephrons. This set of results is consistent with a pattern of partial synchronization (Pikovsky et al., 2001; Dahms et al., 2012; Pecora et al., 2014; Steur et al., 2016; Su et al., 2019).

Analysis of the myogenic oscillation revealed interaction patterns different in various aspects from the TGF waveforms. Results from the nephron pairs, 2 v. 3, 6 v. 7, and 9 v. 10 were similar in the myogenic and TGF dynamics: synchronization metrics close to 1.0 and phase angle differences close to 0. For all interactions involving unpaired nephrons the synchronization metrics had lower values in the myogenic than in the TGF dynamics, and the phase angle differences were larger.

From the same run displayed in Figure 3; Figure 5 displays time series of blood pressure in the 9 nodes of the arterial network. Tables 3, 4 contain the synchronization metrics and phase angle differences for the TGF and myogenic oscillations. Compared with the data shown in Tables 1, 2, the synchronization metrics in these nodal pressure calculations show partial synchronization for both the TGF and myogenic oscillations and that the extent of the synchronization is inversely proportional to the distance between the nodes.

Blood flow into all nephrons is periodic because of the actions of TGF and the myogenic mechanism operating in each afferent arteriole. The periodic withdrawal of blood into upstream afferent arterioles introduces periodicities into the blood flow in the arterial segments supplying downstream arterioles. The periodic variation of arteriolar hydraulic conductances may or may not be synchronized with the periodic flow arriving from upstream regions of the network, and the vascular pressures in the arterial nodes will therefore depend on the specific structural details of each region. Table 4 contains the synchronization metrics and phase angle differences between the hydrostatic pressure in each tubule and the arterial pressure in the nodes from which their arterioles arise. The synchronization metrics (Columns **A** and **C**)are higher for the strongly coupled nephron pairs than for the other nephrons, and the same is true for the phase angle differences. The increased synchronization metric of strongly coupled nephrons with the arterial nodes supplying them is therefore likely the result of the increased signal strength emerging from the strong coupling.

## Discussion

Experimental results reveal the presence of proximal tubular hydrostatic pressure oscillations at 2 distinct frequencies in individual nephrons of rat kidneys. The oscillations reflect the operation of 2 separate mechanisms regulating blood flow into each afferent arteriole and glomerular filtration into its associated nephron. Both mechanisms act on ionic permeabilities of arteriolar smooth muscle cells, interact with each other, and generate a bimodal electrical signal that propagates along arterial segments to neighboring arterioles. The arteriolar oscillations synchronize fully if the arteriolar sites of origin are from the same artery, but not otherwise. The experimental observations supporting these conclusions were all made on nephrons at the renal surface. Most nephrons lie below the surface, and are therefore not available for measurement *in vivo*. We have now developed a model of a network of arteries, arterioles, and nephrons to determine the extent of synchronized nephron fields, and the conditions that affect synchronized field formation.

The model was formed from a model of single nephrons and their arterioles Marsh et al. (2005a) that was later expanded to 2 nephrons (Marsh et al., 2009; Laugesen et al., 2010), and then to 24 (Marsh et al., 2013). This last model used an idealized symmetric representation of the arterial network, with all its nephrons in 2 nephron pairs on the renal surface. We later performed computed tomography on a vascular cast of a rat kidney, and found variable arterial branching patterns, and afferent arteriolar origins conforming to 3 different motifs. The network model in this paper reproduces each of the 3 motifs Marsh et al. (2017). New features of this model are the presence of variable lengths of arterial segments and the different patterns of arteriolar origins.

The afferent arteriole-nephron model has been validated against experimental results, and we have used it in this work as originally presented. The new problem to be solved is determining the dimensions of the arterial components of the network. The length of arterial segments separating adjacent afferent arterioles follows a Poisson distribution (Postnov et al., 2016; Marsh et al., 2017), which we reproduce with a random number generator. The dimension question then becomes solving the system for the radius of each arterial segment in the model. These segments lie entirely below the renal surface and, to the best of our knowledge, no measurements of their dimensions have been made *in vivo*.

We used the network model to predict the radius of each arterial segment. The hypothesis that forms the basis for our approach is that nephrons and their afferent arterioles exercise all regulatory activity affecting blood flow, and that the arteries maintain constant radii over epochs of 2–3 h. There is abundant evidence for the operation of both TGF and the myogenic mechanism, and no evidence for participation of intra-renal arteries in the regulation of blood flow to individual afferent arterioles. Starting from an arbitrary set of initial conditions, we calculated the vascular pressure in each arterial node, applied Murray’s Law to adjust the arterial diameters after each of a series of iterations, and continued the process until the Euclidian norm of the vector of arterial node pressures reached an asymptote. Murray’s Law was originally derived to calculate the minimum energy required to deliver steady laminar flow from a single vessel that bifurcates into 2 identical branches Murray (1926). The law predicts the diameter of the two branches needed to achieve a minimum energy loss for a time-invariant fluid flow through the bifurcation. We make no claim that the law is applicable to a network with active afferent arterioles serving as boundary conditions. We used Murray’s law to perturb the diameters of all the arterial branches to allow the individual nephron-afferent arteriolar units to exchange information until further iterations could no longer change the vascular nodal pressures.

Arteries are adaptive structures with a variety of mechanisms available to adjust their radii. Some of these mechanisms will respond to local changes in pressures, flows, shear stress, and vasoactive agents released locally or elsewhere into the circulation, and still others will operate under genetic or epigenetic control, and each will act within its own dynamic limits. We simply assume that the arteries will respond to changes in the vascular pressures and flow and the entire system will relax to a more or less stable state for epochs of 2–3 h, the duration of a typical experiment whose results form the basis for this modeling effort, and a period of time during which the mean arterial blood pressure in conscious animals follows a 1/f distribution (Marsh et al., 1990; Wagner and Persson, 1994; Holstein-Rathlou et al., 1995).

The model reproduces a number of interactions: TGF and the myogenic mechanism within vascular smooth muscles, leading to modulation of amplitude and frequency of the myogenic mechanism by TGF; bimodal electrical signals generated by the same vascular smooth muscle cells and propagated into the electrically conductive arterial segments; and periodic withdrawal of blood flow from the upstream arterial flow, imposing periodicity on the vascular pressures in the arterial nodes. This system of interactions represents a high-order system generating its dynamics in a specific network structure imposed by the functional needs of nephrons.

Tree structures, a category that includes the nephron-vascular network we found Marsh et al. (2017), are described in graph theory as having N vertices and N-1 edges, where the edges are arterial segments connecting one vertex with another, and the vertices are afferent arterioles and the nephrons they supply. The nephron-vascular network is a rooted tree, in which one vertex is designated as the tree root. In our study of renal vascular structure we examined more than 1,100 glomeruli and did not find any supplied by more than a single afferent arteriole, an observation consistent with the definition of a tree network structure. Random networks, another and well studied different network structure, have no limits on the number of connections nodes may have, and this freedom allows large hubs to emerge. The renal vascular network has no hubs. Effective renal function requires a matching of axial nephron flow to epithelial transport capacity, a condition that would be difficult to satisfy in the mammalian kidney with a random vascular network.

Mammalian kidneys serve to regulate the volume and composition of the extracellular fluid of the body. Filtration in the glomerulus of each nephron creates tubular fluid that flows along the tubule under a gradient of hydrostatic pressure. The Reynolds’ number is low and the Peclet number is high, tubular fluid flow is laminar, and epithelial transport processes reabsorb water and a variety of solutes, returning them to the peritubular capillaries and thence to the systemic circulation. The tubular fluid flow rate remaining at the macula densa is about 5%–10% of the glomerular filtration rate. The epithelial transport processes responsible for maintaining the volume and composition of the extracellular fluids of the body operate downstream of the macula densa, they have limited dynamic ranges, and regulating the delivery rate of the fluid flow and the mass flow of ionic components, chiefly Na^+^, K^+^, and Cl^−^, requires effective feedback regulation, a function provided by TGF.

In its current form, the model simulates the behavior of the various components. Afferent arterioles originating from the same site on terminal arterial segments - nephrons 2 and 3, 6 and 7, and 9 and 10 - formed fully synchronized pairs that are present in each of the 15 simulations and that remain synchronized for the duration of the simulation. Each of these afferent arterioles is in direct contact with their pair mate, forming a strong coupling. Each pair constitutes a cluster, but each cluster is not necessarily synchronized with the others. The other 4 afferent arterioles in the model are separated from each other and from the strongly coupled pairs through lengths of electrically conductive artery, the connection representing a form of weak coupling. The interactions involving these weakly coupled nephrons produce synchronization metrics less than 1.0, and variable phase angle differences. Thus, one major result of the study is the finding that not all nephrons behave identically, despite the fact that they all interact, a condition referred to as partial synchronization or cluster synchronization. Analysis of systems whose dynamics conform to this description have attributed its origin to coupling with delays (Dahms et al., 2012; Steur et al., 2016; Su et al., 2019); to variable coupling strength Pikovsky et al. (2001); and to symmetries and symmetry breaking in the network structure Pecora et al. (2014). The weak couplings lead to variable phase angle differences, as shown in Tables 1 B and 2 B, and the variable phase differences form variable delays that are the most likely causes of partial synchronization in the nephron-arterial network. Connection strengths are also variable. Clusters form and persist when afferent arterioles are directly coupled at the end of terminal arteries. The interposition of electrically conductive arterial segments produce conductances that vary with both the length and radius of the segment, and that form a set of weak couplings. We did not assess the symmetries of the arterial network, but there is no reason to doubt that the approach taken by Pecora et al. (2014) could be useful.

Interactions among the nephrons in the network lead to oscillations with variable period lengths longer than those of the TGF oscillation in any of the nephrons. This result is consistent with experimental results (Pavlov et al., 2008; Siu et al., 2009; Brazhe et al., 2014). These longer oscillations occur in each of the 15 simulations. They are not strictly periodic, they occur in different regions of the model structure in different simulations, they are not commensurate with the TGF oscillations, and they are compatible with the definition of quasiperiodicity. We interpret this result to signify that the slower oscillations are the result of interactions among elements in the network, and reflect the fact that these combinations of nephrons, arterioles, and arteries do not become fully synchronized. We speculate that this is likely a persistent behavior that does not depend on additional factors such as external pacemakers, or extensions of the network to include additional nephrons.

The structural details of a network are often important in determining its dynamics. The kidney provides examples of this principle. Table 1–3 show average values and standard deviations of the synchronization measures from 15 simulations, each different from the others because a unique seed applied to a random number generator varied both nephron and arterial lengths. The variation in the output from one simulation to the next is due to the induced structural variation in the basic model. Additional variation in the placement of each afferent arteriole, a strategy we did not implement, will induce further variation in the dynamics. The model simulates the dynamics of 10 nephrons, while the rat kidney has around 40,000. The proximal and distal tubules of all nephrons are contained within the renal cortex, are convoluted, and have no preferred orientation with respect to the major axes of the organ. The segments intermediate between the proximal and distal tubules, the descending and ascending limbs of the loops of Henle, operate under different geometric arrangements. They run parallel to an axis extending from the midline of the cortical surface to the furthest extreme of the inner medulla. This anatomical arrangement represents a form of symmetry breaking, and additional symmetry breaking arises from the segregation of tubular structures and blood vessels into separate compartments in the medulla Wexler et al. (1991). This countercurrent flow structure is a critically important feature of the mechanism that produces hypertonic urine.

Blood flow in many organs is adjusted to maintain local blood gas concentrations. The kidney is an exception. Venous $p_{O_{2}}$ in the kidney is one of the highest of any organ in the body because of the high blood flow needed to provide a glomerular filtrate from the plasma of the blood perfusing the organ. The result is that tubules in the renal cortex receive an ample supply of O_2_ to meet their energy needs. Thick ascending limbs of Henle’s loop reabsorb Na and Cl, an active process requiring O_2_ supplied by blood flowing into the medulla from the cortex. The operation of an O_2_ consuming process distributed along the axis of the medulla creates an axial gradient of $p_{O_{2}}$ with a maximum in the renal cortex and a minimum at the tip of the renal papilla, the furthest extreme of the inner medulla. Schurek and Johns measured the $p_{O_{2}}$ over the surface of a rat kidney *in vivo* and found an oscillation at the same frequency as that of the TGF oscillation Schurek and Johns (1997). The oscillating flow of tubular fluid in the thick ascending limb will provide an oscillating load of NaCl for epithelial transport, and the oscillation of $p_{O_{2}}$ indicates that the energetics of epithelial transport could become rate-limiting on the production of the signal that is thought to regulate afferent arteriolar diameter.

A feature of the network worth noting is the signal pathways it provides between afferent arterioles of surface nephrons and those that lie below the surface. The arteries supplying surface nephrons, represented in the model as numbers 6 through 10, are at the downstream end of each network tree, and will therefore have the lowest arterial pressures in the tree. Figures 5, 6 show this distribution of pressures in the arterial nodes. The decline of pressure with distance is the effect of frictional energy loss. Interactions mediated through the network admit signals from the surface nephrons to the upstream components to make adjustments that increase pressure downstream, and they also send signals from the upstream nephrons to support downstream nephron dynamics. Nephrogenesis, the formation process of nephrons and their vascular network, is complete in the post-natal period, and while nephron size may vary in the lifetime of the organism, nephron number thereafter can only decrease. Low blood pressures can lead to hypoxia and tubule death. Effective renal function over the life of the organism therefore requires a network structure that retains its topological and interactive characteristics over major fractions of its lifetime.

Finally, the experimental result that motivated this study was the observation that paired nephrons synchronized if their afferent arterioles arose from a common artery. We asked whether larger synchronized nephron clusters might exist when more nephrons were included in a network whose structure contained measured details. The answer we found to this question is neither yes nor no, but partial synchronization, an intermediate state. We suggest that the strongly coupled nephron pairs together with the weakly coupled and partially synchronized nephron group provide a more flexible and adaptive aggregate capable of an appropriate response to a greater variety of challenges than would be possible with a more rigid completely synchronized structure.

## Data Availability

The datasets presented in this study can be found in online repositories. The names of the repository/repositories and accession number(s) can be found in the article/Supplementary Material. Computer programs are available at: https://github.com/donaldjmarsh/nephron-artery.
