# Supplementary material for: Interacting information streams on the nephron arterial network
Source: Front Netw Physiol. 2023 Oct 19;3:1254964. doi: 10.3389/fnetp.2023.1254964 (PMC10620968; doi:10.3389/fnetp.2023.1254964)
Supplement: Supplementary file 1 [file DataSheet1.PDF]

Supplementary Material

The material in this supplement is reproduced from Marsh et al. (2005), Marsh et al. (2009), and Marsh et al. (2013). The material includes previously published Glossary, model equations, and model parameters.

GLOSSARY

Subscripts

A	Afferent arteriole
E	Efferent arteriole
GC	Glomerular capillary
IN	Internephron
I	Interstitial
S	NaCl
T	Renal tubule

Independent Variables

t	Time, s
z	Tubule position, cm
z_{GC}	Glomerular capillary position, cm

Dependent Tubular Variables

$C_s(z,t)$	NaCl concentration, mM
$P_{z,t}$	Pressure, mmHg
$Q_{z,t}$	Flow, cm^3/s

Dependent Glomerular Capillary Variable

$C_{z_{GC}}$	Glomerular capillary protein concentration, g/l

Auxiliary Variables

$J(z,t)$	Solute (mmmol/min) or volume (nl/min) flux
$R(t)$	Vascular resistance, dyn·s·cm^{-5}
$r(z,t)$	Tubular radius, cm

Dependent Arteriolar Variables

$Ca_\imath(m_\infty, \nu, t)$	Intracellular Ca concentration , mM
$n(\nu, Ca_\imath, t)$	Fraction of open K channels
$\nu(Ca_\imath, n, t)$	Membrane electrical potential difference, mv
$x(P_A, t)$	Length of parallel elastic element, cm
$y(\xi, \omega, t)$	Length of the contractile element, cm
$\omega(\xi, t)$	Fraction of actual: possible actin-myosin cross bridges

Auxiliary Arteriolar Variables

$m_\infty(\nu)$	Equilibrium distribution of Ca open-channel states
$n_\infty(\nu, Ca_\imath)$	Equilibrium distribution of K open-channel states
$u(x, y)$	Length of series elastic component, cm
$\xi(Ca_\imath)$	Fraction of phosphorylated myosin light chain

MODELS

TGF and the Tubule Model

The equations describing pressure, P_T, and flow, Q_T, for a non-compressible Newtonian fluid in a compliant, reabsorbing tubule at low Reynolds number are:

$$\frac{\partial P_T}{\partial z} = -\frac{\rho}{\pi r^2}\frac{\partial Q_T}{dt} - \frac{8\eta}{\pi r^4}Q_T, \qquad 0 \leq z \leq Z, t \geq 0 \tag{S1}$$

and

$$\frac{\partial Q_T}{\partial z} = -2\pi r\frac{\partial r}{\partial P_T}\frac{\partial P_T}{\partial t} - J_v(z), \qquad 0 \leq z \leq Z, t \geq 0 \tag{S2}$$

where ρ is the tubular fluid density and η its viscosity.

The boundary conditions for Eqs. 1 and 2 are the tubular inflow rate, $Q_{T(0)}$, calculated from the equations of the glomerular model, and the tubular outlet pressure, estimated from experimental measurements.

$$P_T(Z) = \frac{Q_T(Z)}{(\alpha_T P_T(Z) + \beta_T)^4} \tag{S3}$$

The proximal tubule, the descending and ascending limbs of the loop of Henle have distinct physiological properties and are each represented separately.

Proximal tubular fluid reabsorption, J_v, is

$$J_v = \kappa \exp(-\theta z) \tag{S4}$$

For the descending limb of Henle's loop, J_v is proportional to the transtubular osmotic pressure gradient

$$J_v = L_p(C_S - C_l) \tag{S5}$$

where L_p subsumes the universal gas constant and the absolute temperature, and has units mol$^{-1}\cdot$ s $^{-1}$.

The ascending limb of Henle's loop is impermeable to water and volume reabsorption in this segment is therefore 0.

Tubular radius is expressed as a linear function of the trans-tubular hydrostatic pressure difference.

$$r(z) = \gamma(P_T(z) - P_I) + r_0 \tag{S6}$$

NaCl is treated as a single solute, s. For a solute s in a reabsorbing tubule with an axial flow, Q_T, mass balance requires that

$$\frac{\partial AC_s}{\partial t} = -\frac{\partial Q_T C_s}{\partial z} - J_S \tag{S7}$$

where A is the tubule cross-sectional area, and C_S is the solute concentration.

The proximal tubule reabsorbs tubular fluid isosmotically. NaCl is the principal osmotically active component. C_S is assumed to be constant along that segment, and Eq. 7 is solved only for the loop of Henle. For the initial condition, C_S is set equal to the interstitial concentration, 150 mM, at the beginning of the descending limb. The first term in Eq. 8 represents passive epithelial transport in the descending limb of Henle's loop. The second term in Eq. 8 characterizes active solute transport with Michaelis-Menten kinetics and the sum of the two terms is used to represent epithelial transport in the ascending limb. Thus,

$$J_s = L_s(C_S - C_I) + \frac{V_{max} C_S}{K_m + C_s} \tag{S8}$$

where $V_{max} = 0$ for the descending limb, where there is no active transport.

The interstitial NaCl concentration, C_I, varies linearly from 150 mM at the corticomedullary boundary to 285 mM at the border between the inner and outer medulla, and by $C_I(z^* + z_D LH_)) = 285 + 20tanh(.023(z^* - z^*_{DLH_0}))$ thereafter. $z^*_{DLH_0}$is the length of cortical tubules from glomerulus to the bend of Henle's loop divided by the spatial step size, and Z^* is the distance from the glomerulus in the additional length of longer descending limbs, divided by the spatial step size. For the cortical thick ascending limb of the loop of Helle, C_I was set to 150 mM.

TGF: afferent arteriolar model. The action of TGF is described by

$$\vartheta = \vartheta_{max} - \frac{\psi}{1 + \exp\left[k(C_s(md) - C_{1/2})\right]} \tag{S9}$$

where ϑ_{max} is the maximum obtainable response, ψ is the dynamic range, $C_s(md)$ is the NaCl concentration in tubular fluid at the macua densa, k is the feedback gain and $C_{1/2}$ is the NaCl concentration that gives the half-maximum response.

Glomerular model. Glomerular capillaries are impermeable to plasma proteins and the glomerular filtrate, $Q_T(0)$, is therefore

$$Q_T = \left(1 - \frac{C_A}{C_E}\right) Q_A \tag{S10}$$

The plasma flow to the glomerular capillaries, Q_A, is

$$\frac{Q_A}{(1-Ht_A)} = \frac{(P_A - P_{GC})}{R_A} \tag{S11}$$

where Ht_A is the hematocrit of arterial blood, P_A is the arterial pressure at the origin of the afferent arteriole, P_{GC} is the hydrostatic pressure in the glomerular capillaries, and R_A is the hydraulic resistance of the afferent arteriole.

Glomerular capillary hydrostatic pressure, P_{GC}, is obtained by assuming that afferent blood flow less the glomerular filtration rate, Q_T, passes through the efferent arteriole and that thereafter the reabsorbed tubular fluid is added to the efferent arteriolar blood flow, which then passes through a distal resistance, R_v to the venous compartment where the hydrostatic pressure is assumed to be zero.

$$P_{GC} = R_E\left[\frac{Q_A}{(1-Ht_A)} - Q_T(0)\right] + \frac{Q_A R_v}{(1-Ht_A)} \tag{S12}$$

The filtration process that causes the change in protein concentration is proportional to the sum of local hydrostatic and oncotic pressure differences

$$\frac{dC}{dz_{GC}} = \frac{K_f}{LQ_AC_A}C^2\left[P_{GC} - P_T(0) - \Pi(C)\right] \tag{S13}$$

where z_{GC} is the fractional position along the gomerular capillary, K_f is the filtration coefficient, and L is the length of an idealized glomerular capillary. The initial condition, $P_{GC}(0)$, is derived from the arterial pressure at the origin of the afferent arteriole, and the calculation of afferent arteriolar vascular resistance, as described below.

The plasma oncotic pressure, $\Pi(C)$, is found from:

$$\Pi(c) = a_{GC}C + b_{GC}C^2 \tag{S14}$$

Myogenic model.

The model of the afferent arteriole is based on Gonzalez-Fernandez and Ermentrout (1994), who developed it for application to cerebral arterioles.The afferent arteriole is modeled as 2 segments in sequence, each segment with a variable hydraulic resistance under the control of both the myogenic mechanism and TGF. The resistances in these segments are given as:

$$R_{A,\jmath} = \frac{\Lambda_\jmath}{r_\jmath^4}, \jmath = 1, 2 \tag{S15}$$

where $\Lambda_\jmath$ combines the segment's length and the blood viscosity. Each of the 2 arteriolar segments is modeled separately as a set 6 ordinary differential equations and their associated constitutive relationships.

Transport of ions and membrane potential. The equilibrium distribution of Ca open-channel states, m_∞, as a function of the membrane voltage, $\nu_\jmath$, of the arteriolar segment $\jmath$ is

$$m_{\infty,j}(\nu_j) = 0.5\left(1 + \tanh\frac{\nu_j - \nu_1}{\nu_2}\right), \quad j = 1,2 \tag{S16}$$

where ν_j is the voltage at which half the channels are open, ν_2 is a measure of the spread of the distribution, and j are the first and second arteriolar segments. This equation is intended to represent a mixture of L- and T-type Ca channels.

For K channels, the distribution is

$$n_{\infty,j}(\nu_j, Ca_i) = 0.5\left(1 + \tanh\frac{\nu_j - \nu_{3,j}}{\nu_4}\right), \quad j = 1,2 \tag{S17}$$

where

$$\nu_{3,j} = -\frac{\nu_5}{2}\tanh\frac{(Ca_{i,\nu_j} - Ca_3)}{Ca_4} + \nu_6, \quad j = 1,2 \tag{S18}$$

Equation 18 provides a Ca-dependent shift in the distribution of K open states with respect to membrane voltage. The time course of the fraction of open K channels, ν, is given by

$$\frac{dn_j}{dt} = \lambda(\nu_j)[n_{\infty,\nu_j}(\nu_j, Ca_{i,j}) - n_j], \quad j = 1,2 \tag{S19}$$

where

$$\lambda(\nu_j) = \phi_n \cosh\frac{(\nu_j - \nu_{3,j})}{2\nu_4}, \quad j = 1,2 \tag{S20}$$

The rate of change of the membrane potential is related to membrane currents by

$$C_A\frac{d\nu_j}{dt} = \begin{cases} -I_{L,j} - I_{K,j} - I_{Ca,j} - I_{IN}, & j = 1 \\ -I_{L,j} - I_{K,j} - I_{Ca,j} - I_{IC}, & j = 2 \end{cases} \tag{S21}$$

where C_A is membrane capacitance, and $I_{L,j}$, $I_{K,j}$, $I_{Ca,j}$ are the leak, potassium, and calcium currents of the$j$th arteriolar segment, respectively, I_{IC} is intersegmental current, and I_{IN} is internephron current. Assuming an ohmic voltage-current relationship

$$C_A\frac{d\nu_j}{dt} = \begin{cases} -g_L(\nu_j - \nu_L) - g_k n_j(\nu_j - \nu_K) - g_{Ca_{j,c}} m_{\infty,j}(\nu_j - \nu_{Ca}) \\ -g_{IN}(\nu_j - \nu_{node}), & j = 1, k = 2 \\ -g_L(\nu_j - \nu_L) - g_K n_j(\nu_j - \nu_K) - g_{Ca,j,c} m_{\infty,j}(\nu_j - \nu_{Ca}) \\ -g_{IC}(\nu_k - \nu_j), & j = 2, k = 1 \end{cases} \tag{S22}$$

where g_L, g_K, g_{Ca}, g_{IC},and g_{IN} are the whole membrane conductances for the leak, K, Ca, intersegmental and internephron currents, respectively, ν_L,ν_K, and ν_{Ca} are the corresponding Nernst potentials, and $g_{Ca,j,c} = (1 + \zeta\vartheta_j)g_{Ca}$. The variable ϑ_j is the input from TGF to the jth arteriolar segment, and ζ is the TGF-myogenic coupling parameter. The coefficient $g_{Ca,j,c}$ therefore represents the membrane Ca conductance coupled to TGF in the jth segment.The rate of change of Ca in the cytosol is given by

$$\frac{dCa_{T,\jmath}}{dt} = -\alpha_A g_{Ca,\jmath,c} m_\infty(\nu_\jmath - \nu_{Ca}) - k_{Ca} Ca_{\imath,\jmath}, \quad j = 1,2 \tag{S23}$$

where $\alpha_A = 1/2\beta_A V_{cell} F$. β_A is the fraction of the total cell volume occupied by the cytosol, V_{cell} is the total cell volume, F is faraday's constant, and $k_C a$ is the first-order rate constant for Ca removal from the cytosol. By assuming that cytosolic free Ca is in equilibrium with various Ca buffers whose total concentration is B_T and that can be represented by a single equilibrium constant K_d one may arrive at

$$\frac{Ca_{\imath,\jmath}}{dt} = -(\alpha_A g_{Ca,\jmath,c} m_\infty(\nu_\jmath - \nu_{Ca}) - k_{Ca} Ca_{\imath,\jmath})\varpi, \quad \jmath = 1,2 \tag{S24}$$

where

$$\varpi_\jmath = \frac{(K_d + Ca_{\imath,\jmath})^2}{(K_d + Ca_{\imath,\jmath})^2 + K_d B_T} \tag{S25}$$

Myosin light chain phosphorylation Activation of smooth muscle is assumed to occur in response to Ca-dependent phosphorylation of myosin light chain. One assumes that the kinetics of phosphorylation are rapid compared with the time scale of the vascular events, and defines ξ as the fraction of myosin light chain sites that are phosphorylated.

$$\xi_\jmath = \frac{Ca_{i,\jmath}^q}{Ca_{i,m}^q + Ca_{i,\jmath}^q}, \quad \jmath = 1,2 \tag{S26}$$

We describe the relationship between phosphorylated light chain myosin and cross bridge formation phenomenologically in terms of a binding distribution. Letting $\omega_\jmath$ represent the fraction formed of the total possible cross bridges in the $\jmath$th arteriolar segment, we arrive at the expression

$$\frac{d\omega_\jmath}{dt} = k_\xi \left(\frac{\xi_\jmath}{\xi_m + \xi_\jmath} - \omega_\jmath \right), \quad \jmath = 1,2 \tag{S27}$$

Wall stress and contraction velocity. The contractile mechanism has length y, the series elastic component length u, and the parallel elastic component length $x = u + y$. The stresses σ normal to the surface of a longitudinal slice, S_A, through the vessel wall and associated with each element are

$$\sigma_x = x_3 \left(1 + \tanh \frac{x^* - x_1}{x_2} \right) + x_4(x^* - x_5) - x_8 \left(\frac{x_6}{x^* - x_7} \right)^2 - x_9 \tag{S28}$$

$$\sigma_u = u_2 \exp(u_1 u / x_0) - u_3 \tag{S29}$$

and

$$\sigma_y = \frac{\omega}{\omega_{ref}} \frac{\exp\left(-\frac{(y/x_0 - y_0)^2}{2s(y/x_0)^2}\right) - y_3}{1 - y_3} \tag{S30}$$

where $x^* = x/x_0$, x_0 is a reference length, $u = (x - y)$, $s(y) = [y_1/(y + y_2)]^{y_4}$, and $\omega_{ref} = \omega(Ca_{i,ref}/(\xi_m + \xi(Ca_{i,ref}))$. The x_{1-9}, u_{1-3} and y_{1-3} are coefficients used to fit the various expressions to data from the literature.

The force-velocity relationship for the contractile mechanism is given by

$$\frac{dy}{dt} = -x_0 \upsilon_{ref} \frac{\xi}{\xi_{ref}} a_A \frac{\left(1 - \frac{\sigma_u}{\sigma_y}\right)}{\left(a_A + \frac{\sigma_u}{\sigma_y}\right)}, \quad 0 \leq \frac{\sigma_u}{\sigma_y} \leq 1$$

or

$$\frac{dy}{dt} = x_0 C_A \left(\exp\left(b_A \left(\frac{\sigma_u}{\sigma_y} - d_A\right)\right) - \exp(b_A(1 - d_A))\right), \quad 1 \leq \frac{\sigma_u}{\sigma_y} \tag{S31}$$

where υ_{ref},a_A, b_A, c_A, and d_A are constants.

The hoop forces f acting on a longitudinal section S_A of the blood vessel wall are given by

$$f_{\Delta P} = \frac{1}{2} \Delta P \left(\frac{x}{\pi} - \frac{A}{x}\right), \quad x > \sqrt{\pi A} \tag{S32}$$

where ΔP is the transmural pressure difference between the vascular wall and the interstitial space, and A is the cress-sectional area of the vascular smooth muscle cells.

$$f_x = w_e S_A \sigma_x \sigma_0 \tag{S33}$$

$$f_u = w_m S_a \sigma_u \sigma_0 \tag{S34}$$

w_e and w_m are weighting factors , and σ_0 is a reference stress.

The rate of change of the circumference $x$is given by

$$\frac{dy}{dt} = \frac{1}{\tau}(f_{\Delta P} - f_x - f_u) \tag{S35}$$

subject to the geometric compatibility condition:

$$x = u + y \tag{S36}$$

PARAMETERS

Table S1. *Tubule parameters*

Parameter	Value
ρ	1 g/cm^3
η	7.2$\times$10^{-3} poise
K_M	20 mM
L_p	5$\times$10^{-2}cm Mol^{-1}s^{-1}
L_S	2.96$\times$10^{-6}cm s^{-1}
P_t	5 mmHg
V$_{max}$	0.652$\times$10^{-7} mmol cm^1 s^{-1}
θ	1.3 cm^{-1}
κ	5.6$\times$10^{-7} nl min^{-1} cm^{-2}

Table S2. *Glomerular parameters*

Parameter	Value
a_{GC}	1.631 $\times$ 10^{-1} mmHg g^{-1}
b_{GC}	2.94 $\times$ 10^{-3}mmHg^2 g^{-2}
C_A	57 g/l
Ht_A	0.5
K_f	2.5 nl min^{-1} mmHg^{-1}
R_E	2.0$\times$10^{-2} mmHg min nl^{-1}
R_ν	7.02$\times$10^{-2}mmHg min nl^{-1}

Table S3. *Arteriolar membrane parameters*

Parameter	Value
ν_1	-22.5mV
ν_2	25.0 mV
ν_4	14.5 mV
ν_5	8.0mV
ν_6	-15.0 mV
Ca$_3$	400.0 nM
Ca$_4$	150.0 nM
ϕ_n	3.0$\times$10^{-1}s^{-1}
ν_L	-70.0 mV
ν_K	-90.0 mV
ν_{Ca}	80.0 mV

Table S4. *Arteriolar wall mechanical properties*

Parameter	Value
x_0	1.5×10^{-1}cm
x_1	1.2
x_2	1.3×10^{-1}
x_3	2.22
x_4	7.12×10^{-1}
x_5	8.0×10^{-1}
x_6	1.0×10^{-2}
x_7	3.21×10^{-1}
x_8	8.9×10^{-1}
x_9	9.0×10^{-3}
u_1	41.8
u_2	4.74×10^{-2}
u_3	5.84×10^{-2}
y_1	6.39×10^{-1}
y_2	3.5×10^{-1}
y_3	7.89×10^{-1}
y_4	8.0×10^{-1}

Table S5. *Arteriolar cell coefficients*

Parameter	Value
C_A	1.96×10^{-14} coulomb/mV
g_L	4.0 pS
g_K	15.99 pS
g_{Ca}	7.13 pS
g_{IC}	8.2 pS
K_d	1.0×10^{3} nM
B_T	1.0×10^{5} nM
α_A	2.24×10^{2} nM/coulomb
k_{Ca}	1.80×10^{2} s^{-1}
ζ	0.255

Table S6. *Arteriolar geometric coefficients.*

Parameter	Value
S	9.0×10^{-3}cm^2
A	1.4×10^{-3}cm^2
w_e	2.5×10^{-1}
w_m	8.5×10^{-1}

Table S7 *Tubuloglomerular feedback parameters*

Parameter	Value
$C_{1/2}$	44 mmol^{-1}
k	0.15 l mmol-1
ϑ_{max}	0.455 mmHg min nl-1
ψ	0.91 mmHg min nl^{-1}

Table S8. *Muscle dynamics*

Parameter	Value
$\mathrm{Ca}_{i,m}$	3.5×10^{2}nM
q	3.0
$\mathrm{Ca}_{i,ref}$	4.0×10^{2}nM
k_ξ	$1.1\times10^{2}\mathrm{s}^{-1}$
v_{ref}	$2.4\times10^{-1}\mathrm{s}^{-1}$
a_A	2.81×10^{-1}
b_A	5.0
c_A	3.0×10^{-2}
d_A	1.3

Table S9. Network Conductances

g_{IN}	5×10^{-1} pS
g_0	3.5×10^{-3} pS

REFERENCES

Gonzalez-Fernandez, J. and Ermentrout, G. (1994). On the origin and dynamics of the vasomotion of small arteries. *Math.Biosci.* 240, 127–167

Marsh, D., Sosnovtseva, O., Chon, K., and Holstein-Rathlou, N.-H. (2005). Nonlinear interactions in renal blood flow regulation. *AmJPhysiol RegulIntegrComp Physiol.* 288, R1143–1159

Marsh, D., Toma, I., Sosnovtseva, O., Peti-Peterdi, J., and Holstein-Rathlou, N. (2009). Electrotonic vascular signal conduction and nephron synchronization. *AmJPhysiol Renal Physiol.* 296, F751–F761

Marsh, D., Wexler, A., Brazhe, A., Postnov, D., Sosnovtseva, O., and Holstein-Rathlou, N. (2013). Multinephron dynamics on the renal vascular network. *Am.J.Physiol.Ren.Physiol.* 304, F88–F102
